# Evaluation and relation of oral health-related quality of life and oral health status in Thalassemia Major patients, a cross-sectional study

**DOI:** 10.1186/s12903-023-03202-9

**Published:** 2023-07-15

**Authors:** Fatemeh Abbasi, Adel Tabesh, Amirmohammad Yavari, Rasoul Makaremi, Omid Bizhani, Mahboobeh Mahmood

**Affiliations:** 1 Dental Research Center, Department of Oral Medicine, Dental research institute, Isfahan University of Medical Sciences, Isfahan, Iran; 2 Student Research Committee, School of Dentistry, Isfahan University of Medical Sciences, Isfahan, Iran; 3 Department of Restorative Dentistry, School of Dentistry, Tehran University of Medical Sciences, Tehran, Iran

**Keywords:** Beta-Thalassemia, Oral health, Quality of life, Oral hygiene index

## Abstract

**Background:**

Beta-Thalassemia is the most common human inherited disease, directly impacting patients’ physical and psychosocial aspects. The present study evaluated oral health status, oral health-related quality of life (OHRQoL), and their correlation in Thalassemia Major patients.

**Methods:**

Two hundred Thalassemia Major patients aged 12–49 participated in this cross-sectional study. Subjects were selected among the patients referred to the Faculty of Dentistry of Isfahan University of Medical Sciences using simple sampling. Oral Health Impact Profile 14 (OHIP 14) was used to assess OHRQoL. The decayed, Missing, and Filled Teeth (DMFT) index was used as an oral health index. SPSS 22 was used for analysis, using T and Pearson Correlation tests.

**Results:**

53% of participants were female, and 47% were male. The mean OHIP-14 score (± SD) was 13.20 (± 7.01). The mean DMFT score was 9.54 (± 5.72). DMFT and total OHIP 14 scores correlated significantly (*p* < 0.001, r = 0.78). All domains of the OHIP-14 score were also significantly correlated with DMFT (*p* < 0.05).

**Conclusions:**

Poor oral health conditions might adversely affect OHRQoL in TM patients. It seems necessary to provide oral treatment needs in order to improve OHRQoL in patients suffering from this particular disease.

## Introduction

Beta-Thalassemia is one of the most common human inherited diseases. Of note, 3% of the human population is a gene vector for the disease. Thalassemia Major (TM), the major form of the disease, causes severe anemia, leading to several medical problems, such as growth and facial deformities. Hattab, in two studies, reported various maxillofacial symptoms in TM patients. These symptoms are mainly the result of excessive bone marrow expansion due to ineffective erythropoiesis [1, 2]. Although proper management has lengthened the patients’ life span, morbidities affect their daily activities and quality of life [3]. Thalassemia directly impacts the physical and psychosocial aspects of patients’ lives. Their oral condition can cause eating, talking, laughing, and sleeping problems. Oral health-related quality of life (OHRQoL) measures the impact of oral status on a patient’s quality of life [4].

The primary means to assess OHRQoL are patient-centered questionnaires, of which the most utilized is Oral Health Impact Profile 14 (OHIP-14) [5]. OHRQoL measurements combined with the classic clinical examination form an established basis to plan the oral-dental treatment for each individual [5, 6]. Thalassemia Major (TM) is a risk factor for dental and periodontal disease and oral infection. It also affects the patient’s face via over-growth of the upper jaw, with concomitant flaring of the anterior maxillary teeth [1, 2]. Therefore, it might deteriorate the patient’s OHRQoL in several aspects regarding oral function. Previously, several studies have evaluated the OHRQoL in children [3, 4, 7, 8] and adults [8] with TM. While some studies reported a correlation between weak oral health and OHRQoL [3], others concluded that there is no such correlation [8]. The present study aimed to evaluate oral health status, oral health-related quality of life (OHRQoL), and their relation in Thalassemia Major patients in Isfahan, Iran.

## Methods & materials

### Study design and participants

This study was approved by the ethics committee of Isfahan University of Medical Sciences (IR.MUI.RESEARCH.REC.1399.651). The ethical guideline for this study was “General Ethical Guidance for Medical Research with Human Participants in the Islamic Republic of Iran.” All methods were performed in accordance with the relevant guidelines and regulations. Written informed consent was obtained from all patients and legal guardians in case of underage.

Two hundred thalassemia major patients participated in this analytic cross-sectional study, conducted in June 2021. According to Mohamadi et al. [9], the sample size was calculated using a standard deviation of 7.63, absolute precision of 1, α = 0.05, and β = 0.2. Patients were all referred to the Oral Medicine Department, Faculty of Dentistry, Isfahan University of Medical Sciences, Isfahan, Iran, for dental check-ups. The study’s goals were explained to each of them by a single researcher, and volunteer patients took part after signing an informed consent form. The patient’s age and gender were recorded.

### OHRQoL assessment

The examiner gave patients a paper sheet on which they answered the OHIP-14 questionnaire. The used version of the questionnaire was valid and reliable for Persian society [5]. All the subjects were literate, and the researcher replied to every question they asked regarding how to answer or understand the meaning of each item so that they could all finish answering the questionnaire.

### Oral examination

Every subject was examined intra-orally to detect Decayed, Missing, and Filled Teeth (DMFT). Two of the researchers were calibrated for examining the patients. DMFT of each patient was calculated by both of them, according to the World Health Organization (WHO) instructions, and the results were attached to each subject’s filled questionnaire [10]. In case of any difference, study’s supervisor (an oral diseases specialist) solved the conflicts. The examination was conducted under the artificial light of a comfortable dental chair, using a disposable dental mirror and an explorer for each patient. The infection control protocol was strictly respected during the examination.

### Grading and statistical analysis

Each answer choice of the OHIP-14 questionnaire got a code: Never: 0, Seldom: 1, Sometimes: 2, Often: 3, and Always: 4. The OHIP-14 score is the sum of all question codes. Therefore, the higher score shows worse OHRQoL. The score for each domain of the questionnaire was calculated as well. The statistical analysis was done via SPSS software version 22. The Pearson correlation test and T-test were utilized, and the significance level was considered 0.05.

### Ethical considerations

The local research ethics committee passed the study, and the examiner had to ethically refer all the patients to the dental clinic for their treatment needs.

## Results

200 thalassemia major patients participated in the study. Among them, 94 (47%) were male, and 106 (53%) were female. They were 12 to 49 years old, with a mean (± Standard Deviation or SD) of 24.68 (± 8.68). The mean OHIP-14 score was 13.20 (± 7.01), and the mean DMFT score was 9.54 (± 5.72). DMFT and OHIP-14 scores were compared using independent T-test. Both scores were significantly higher in female subjects. The mean scores of OHIP-14 and DMFT for men and women are shown in Table 1.

**Table 1:** Mean OHRQoL and DMFT scores in male and female patients

1. Gender	OHIP-14 score (± SD)	DMFT Mean (± SD)
Male	11.35 (± 6.84)	8.57 (± 5.69)
Female	14.84 (± 7.26)	10.40 (± 5.62)
*p*-value	0.0006^*^	0.023^**^

^*^p-value < 0.001

^**^p-value < 0.05

Pearson correlation test showed a significant correlation between the OHIP-14 score and the age of the patients (*p* < 0.001, r = 0.62). Also, there was a stronger correlation between DMFT and OHIP-14 score (*p* < 0.001, r = 0.78). Scores of all domains of the OHIP-14 questionnaire were correlated to DMFT (*p* < 0.05).

## Discussion

Patients with systemic diseases face various problems, including oral and dental problems, which are less attended to. Oral problems of these patients can cause significant decreases in their quality of life. Recently, evaluating oral health-related quality of life in patients with systemic diseases has been taken into consideration. The importance of the subject grows with the prevalence of the disease. Diseases such as diabetes have been evaluated in many studies, but there are still many diseases to evaluate. Among them are blood disorders such as beta-thalassemia. Thalassemia Major (TM), the major form of the disease, is a potentially life-threatening disease that involves the patient with health morbidities as soon as birth. This huge burden may force the patient or society to ignore oral health conditions. This may be specifically bold in developing countries, where health funds may need to provide complete care for people. Thus, the patients may experience lower life quality because of oral health status, regardless of their life span. Beta-Thalassemia is prevalent in a region known as the Thalassemia Belt. Iran is a country located in this region. It is estimated that 4% of the people of Iran carry the gene of this disease [11]. This adds to the importance of this subject. The present study evaluated oral health status and oral health-related quality of life (OHRQoL) in TM patients.

The present study’s mean age and DMFT score were similar to the study of Motallebnejad et al. [8], While the DMFT score was higher than the studies of Kalbassi et al. [12], Elangovan et al. [13], and Al-Raeesi et al. [14], and lower than the study of Singh et al. [15]. As many of the TM patients in these studies are teenagers, their dental state might have been influenced by oral hygiene care programs in different countries, explaining various DMFT scores. Higher DMFT scores can be a result of less attention to oral hygiene. Also, bone deformities or physical disabilities can challenge oral hygiene maintenance. In such cases, a trained person must perform oral hygiene activities, like brushing and flossing. These patients should be examined regularly by a dentist, so visiting a dentist must be a part of their plans.

The present study found a statistically significant relationship between DMFT and total and all domain scores of OHIP-14, which is similar to the results of Amirabadi et al. [3] and Ebeid et al. [16]. Higher scores of DMFT in TM patients may lead to more problems in their life. The most critical problem with decayed teeth is the pain, which disturbs the patients’ peace. Also, such a situation can prevent the patient from eating, drinking, and sleeping, which are very important in patients with systemic diseases. Considering all these consequences, having regular oral examinations is crucial. Motallebnejad et al. found that oral health status affects psychological aspects of OHRQoL more than physical aspects in TM patients [6]. Physical domains of OHRQoL may be fulfilled by social insurance and health services, while psychological fields may be best provided by familial emotional support, especially in Eastern societies. Both physical and psychological aspects are lacking in the present study. This result reveals insufficient attention to TM patients in different aspects of their lives. These shortcomings reveal the necessity of making health-improving plans for these patients.

One of the study’s limitations was using OHIP-14, a general questionnaire not specialized for a specific disease or age group. All of the patients in this study were assessed with this questionnaire. Developing a new questionnaire can be challenging, and future studies are encouraged to develop more specialized questionnaires for specific age groups. This is especially beneficial for young and old patients because of their critical needs in both health and life. Of course, by developing disease-specific questionnaires, future studies can more precisely detect OHRQoL in TM. Another limitation of the present study is that it just evaluated the patients with thalassemia major. Although the scope of this study includes only thalassemia major patients, having other groups, including healthy people or patients of other diseases, can provide more information and let us make comparisons which give us better vision. This is also recommended for future studies. Many systemic diseases, such as autoimmune diseases or cancers, are suitable for evaluation in the future.

## Conclusion

The present study evaluated both oral health status and oral health-related quality of life in Thalassemia Major patients. This study concluded that poor oral health has a strong correlation with low quality of life, emphasizing the need for attention to oral health in this group of special-care people.

## Data Availability

The data gathered and analyzed in the current study are kept private due to the preference of the authors but will be available from the corresponding author on reasonable requests.

## List of abbreviations

DMFT: Decayed, Missing, and Filled Teeth
OHIP-14: Oral Health Impact Profile 14
OHRQoL: Oral Health-Related Quality of Life
TM: Thalassemia Major
WHO: World Health Organization
